# Acquired antimicrobial resistance genes in *Bordetella* Species: a global genomic analysis

**DOI:** 10.1093/jac/dkaf418

**Published:** 2025-12-01

**Authors:** Biao Tang, Xiaohe Hu, Yu Song, Xu Liu, Guoping Zhao, Min Yue

**Affiliations:** Key Laboratory of Systems Health Science of Zhejiang Province, School of Life Science, Hangzhou Institute for Advanced Study, Hangzhou, University of Chinese Academy of Sciences, China; Key Laboratory of Systems Health Science of Zhejiang Province, School of Life Science, Hangzhou Institute for Advanced Study, Hangzhou, University of Chinese Academy of Sciences, China; Key Laboratory of Systems Health Science of Zhejiang Province, School of Life Science, Hangzhou Institute for Advanced Study, Hangzhou, University of Chinese Academy of Sciences, China; Key Laboratory of Systems Health Science of Zhejiang Province, School of Life Science, Hangzhou Institute for Advanced Study, Hangzhou, University of Chinese Academy of Sciences, China; Key Laboratory of Systems Health Science of Zhejiang Province, School of Life Science, Hangzhou Institute for Advanced Study, Hangzhou, University of Chinese Academy of Sciences, China; National Genomics Data Center & Bio-Med Big Data Center, CAS Key Laboratory of Computational Biology, Shanghai Institute of Nutrition and Health, Chinese Academy of Sciences, Shanghai, China; Department of Microbiology, School of Life Sciences, Fudan University, Shanghai, China; Key Laboratory of Systems Health Science of Zhejiang Province, School of Life Science, Hangzhou Institute for Advanced Study, Hangzhou, University of Chinese Academy of Sciences, China

## Abstract

**Background:**

The genus *Bordetella* comprises Gram-negative pathogens, notably *B. pertussis*, which causes whooping cough, underscoring the need to characterize antimicrobial resistance (AMR) within this group. However, reports of acquired antimicrobial resistance genes (ARGs) in non-*B. pertussis Bordetella* species remain scarce.

**Methods:**

Non–*B. pertussis Bordetella* genomes were retrieved from NCBI, adapters trimmed and low-quality reads filtered, then assembled for analysis. ARGs were identified with Abricate, ResFinder, and BLAST, and their genomic contexts visualized using Easyfig. Core-genome SNP phylogeny and Bayesian clustering delineated lineages, and SNP-based thresholds quantified transmission.

**Results:**

Across 746 genomes, 105 ARGs were identified in three non-*B. pertussis* species: *B. bronchiseptica* (96 ARGs), *B. trematum* (7 ARGs) and *B. avium* (2 ARGs). Ten distinct ARG types covering sulphonamide (*sul1*, *sul2*), tetracycline (*tet*(A), *tet*(G), *tet*(31)), aminoglycoside (*aac(6′)-IIa*, *aadA2*, *aph(3″)-Ib*, *aph(6)-Id*) and β-lactam (*bla*_OXA-2_) resistance were observed. Core-genome SNP analysis of 238 *B. bronchiseptica* strains resolved three lineages, of which lineage 2 harboured the greatest diversity of multidrug-resistant isolates. Transmission-event quantification using SNP thresholds revealed frequent international and cross-host spread, notably human-dog/rabbit transfers and international dissemination between France and the United States.

**Conclusions:**

These findings underscore the importance of monitoring AMR in *Bordetella* species to anticipate and mitigate the spread of resistant strains.

## Introduction

*Bordetella* is a genus of Gram-negative bacteria, nearly all species of which can cause clinical infections in humans. The most commonly implicated species include *B. pertussis*, *B. parapertussis*, *B. holmesii*, and *B. bronchiseptica*.^1^  *B. pertussis*, the causative agent of pertussis (whooping cough), leads to severe acute respiratory infections. In recent years, other *Bordetella* species have also been increasingly reported in clinical cases.^2^ Given this trend, surveillance of antimicrobial resistance (AMR) in *Bordetella* spp. is crucial. Previous studies suggest that *Bordetella* spp. possess relatively conserved genomic sequences, which limit their ability to acquire antimicrobial resistance genes (ARGs) through horizontal gene transfer (HGT), particularly in *B. pertussis*.^3^ Our recent analysis corroborates these findings: among 8579 *B. pertussis* strains analysed, only 22 (0.26%) were predicted to carry ARGs, with the true frequency likely lower due to potential contaminant sequences.^4,5^ However, it remains unclear whether *B. pertussis* could acquire additional ARGs in the future.

HGT is more common among closely related bacterial species,^6^ raising concerns that other *Bordetella* species may serve as intermediaries, acquiring ARGs from external sources and potentially transmitting them to *B. pertussis*. However, the prevalence and genetic context of ARGs in *Bordetella* spp. (excluding *B. pertussis*) remain underexplored. Previous studies have reported the presence of acquired ARGs in *B. bronchiseptica*, for example in the works of Kadlec^7^ and Zhang^8^  *et al.*, which were primarily based on PCR detection. These studies identified genes such as *aac(6’)-Ib*, *sul*, and *tet*.^7,8^ In contrast, reports of resistance genes in other *Bordetella* species are limited, leaving the broader distribution and evolutionary dynamics of ARGs within this genus largely unknown. To address this gap, we systematically investigated the distribution of ARGs in non-*B. pertussis Bordetella* species. This will provide critical insights into the potential risk of ARG acquisition in *B. pertussis* and inform strategies for managing AMR within the *Bordetella* genus.

## Methods

### Genome acquisition and quality control

Whole-genome sequences of *Bordetella* species analysed in this study were obtained from the NCBI database. We retrieved all available assembled genomes and high-throughput sequencing data as of October 28, 2024, selecting non-*B. pertussis* genomes (*n* = 1110). For 709 high-throughput sequencing datasets, we used Trimmomatic (v0.39; parameters: NexteraPE_TruSeq3-PE-2.fa:2:30:10:2:True SLIDINGWINDOW:4:15 LEADING:3 TRAILING:3 MINLEN:36) to remove adapters and low-quality sequences. The cleaned reads were assembled into genome contigs using SPAdes (v4.0.0; parameters: –isolate -k 21,33,55,77,99,127). We performed quality filtering of the 1110 genome sequences by ensuring the uniqueness of the corresponding BioSample. A total of 746 high-quality genomes were retained for downstream analyses based on four quality metrics: genome size, GC content, number of contigs, and N50 value. (Figure S1, available as Supplementary data at *JAC* Online).

### Genome analysis

**Figure 1.**
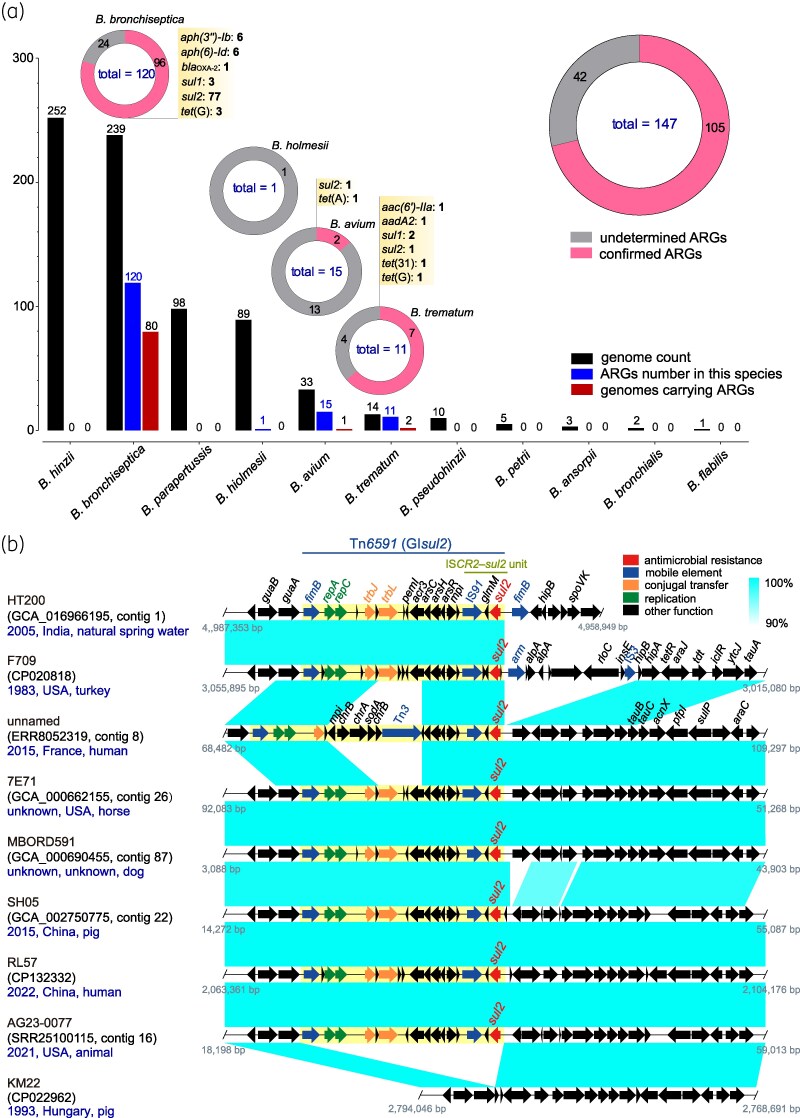
Analysis of acquired ARGs in the *Bordetella* genus and the genetic environment of the *sul2* gene. (a) Prevalence of acquired ARGs in *Bordetella* species. (b) Genetic environment of the *sul2* gene.

Of these, 329 were retrieved from NCBI assemblies, and 417 were obtained from SRA database (Table S1), which includes data from 11 *Bordetella* species (Figure 1a). The Table S1 also includes metadata from the database, with genomic source information validated and supplemented through relevant publications. To identify acquired ARGs, we used Abricate v1.0.1 (https://github.com/tseemann/abricate) and further analysed sequences using ResFinder 4.6.0 (http://genepi.food.dtu.dk/resfinder) and BLAST. Gene context for linear comparisons was visualized using Easyfig v2.2.3.

### Phylogenetic analysis

The genomic sequences of the *B. bronchiseptica* were compared with the reference genomes NCTC10543 (GCA_900636925.1) using Snippy v4.3.6 (https://github.com/tseemann/snippy) with default parameters. A maximum-likelihood (ML) phylogenetic tree was inferred using IQ-TREE v1.6.12^9^ under the best-fit substitution model (selected by ModelFinder) with 1000 bootstrap replicates to assess branch support. Putative recombination regions were identified and removed using Gubbins v3.2.1.^10^ Population structure was further analysed through hierarchical Bayesian clustering implemented in RhierBAPS v1.0.1,^11^ which partitions strains into genetically distinct subgroups based on allelic variation. The phylogenetic tree was visualized and beautified using the online tool iTOL (https://itol.embl.de/).^12^

### Inferring transmission events

To quantify the frequency of transmission events within and between different sources, we aggregated data over all *B. bronchiseptica* genomes and identified transmission events using a threshold-based approach based on SNP distances using a mapping procedure. SNPs were detected in the strain genome using snp-dists v.0.7.0 (https://github.com/tseemann/snp-dists).

## Results and discussion

### The prevalence of ARGs in non-*B. pertussis Bordetella* species

Among the 746 genome sequences, we identified 147 ARG copies across four *Bordetella* species: *B. bronchiseptica* (120), *B. avium* (15), *B. trematum* (11), and *B. holmesii* (1) (Figure 1a). However, due to the potential for exogenous sequence contamination, these numbers may not fully reflect the true ARG distribution. To solve this, we analysed the upstream and downstream sequences of the detected ARGs to exclude contamination, identifying 105 acquired ARGs likely to be genuinely present within the *Bordetella* genome. We confirm that three *Bordetella* species in this study harbour acquired ARGs: *B. bronchiseptica*, *B. avium*, and *B. trematum*. The prevalence of ARGs in these species was 33.6% (80/238), 3.0% (1/33), and 14.3% (2/14), respectively. Across these genomes, we identified 10 distinct ARG types spanning four antimicrobial classes. Sulphonamide resistance genes, particularly *sul2*, were the most prevalent, detected in all three species and found in 79 isolates, making it the dominant resistance genotype. Tetracycline resistance genes, such as *tet*(G), were also common, suggesting that the use of antibiotics and growth promoters in livestock and aquaculture may be key drivers of ARG acquisition in *Bordetella*. Aminoglycoside resistance genes exhibited the highest diversity, including *aac(6’)-IIa*, *aadA2*, *aph(3'’)-Ib*, and *aph(6)-Id*, while β-lactam resistance genes were rare, with *bla*_OXA-2_ detected only once.

It is important to emphasize that the in silico prediction of ARGs does not necessarily equate to their functional expression in *Bordetella* or the manifestation of a corresponding resistance phenotype. Multiple factors, including gene silencing, transcriptional regulation, and synergistic interactions, may modulate the phenotypic outcome. Therefore, comprehensive phenotypic validation of AMR is essential in future research.

### The prevalence of the *sul2* and *tet*(G) genes

The *sul2* gene was most frequently observed in *B. bronchiseptica*. Analysis of its genetic environment suggests that its dissemination is likely driven by the mobile element Tn*6591* (GIsul2) (Figure 1b), which serves as the core region. This element has been detected in *B. bronchiseptica* strains from multiple countries and diverse isolation sources, indicating its widespread presence.

**Figure 2.**
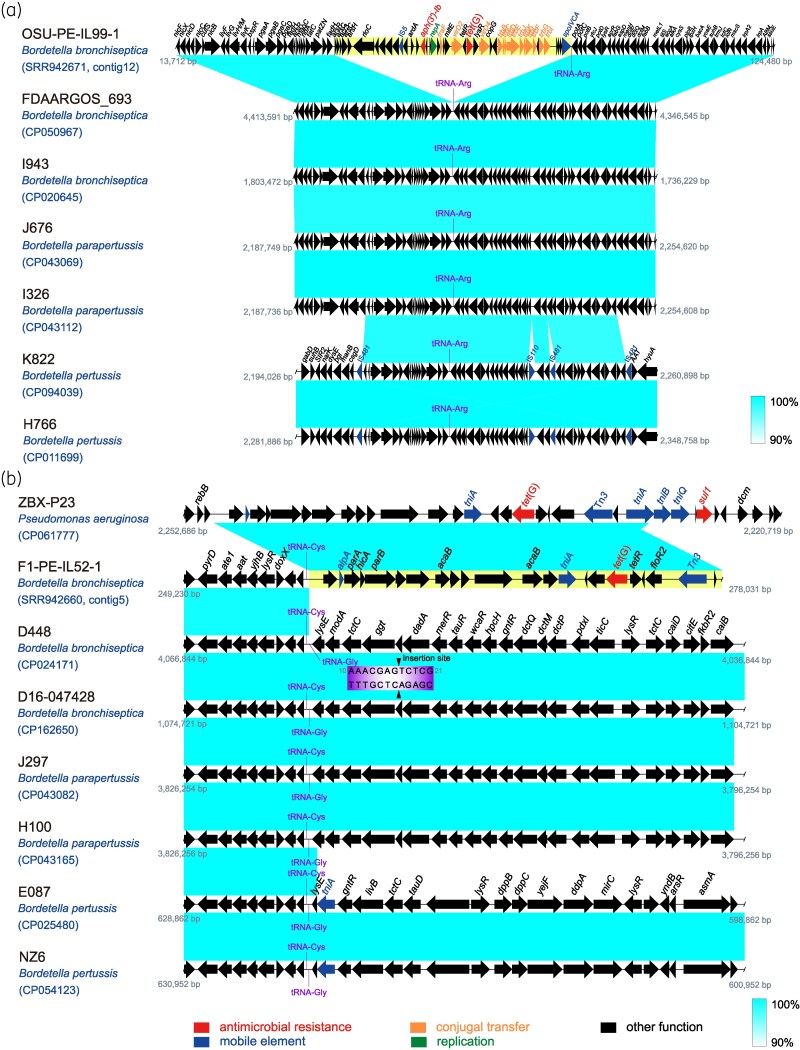
Analysis of the genetic environment of the *tet*(G) gene in *Bordetella.* (a) Schematic of the integrative conjugative element harbouring *tet*(G) and other resistance genes inserted into the *B. bronchiseptica* genome. (b) The *tet*(G) gene and *P. aeruginosa* genomic sequences exhibit a shared genetic context.

For *tet*(G) in *B. bronchiseptica*, we identified two distinct genetic environments (Figure 2). In strain OSU-PE-IL99-1, *tet*(G) is embedded within a ∼44 kb gene fragment, with transfer likely mediated by a site-specific recombinase (Figure 2a). This fragment contains conjugative transfer-associated genes, and the presence of *repA*, a replication gene, suggests a plasmid origin. A tRNA-Arg sequence near the insertion site, known to be associated with genomic island (GI) formation and HGT,^13^ further supports this hypothesis. Additionally, highly homologous gene fragments containing the insertion site were detected in other *Bordetella* species, suggesting that *tet*(G) may be acquired through a similar mechanism in *B. pertussis* and other *Bordetella* species. In strain F1-PE-IL52-1, the *tet*(G) insertion site is located within tRNA-Gly and adjacent to tRNA-Cys (Figure 2b). Due to contig size limitations, part of the foreign fragment could not be definitively mapped to *Bordetella* sequences. Comparative analysis suggests that its transfer may be mediated by transposase, such as Tn*3* or *tniA*, with the fragment likely originating from the *Pseudomonas aeruginosa* chromosome. Furthermore, homologous sequences containing the insertion site were detected in other *Bordetella* species, underscoring the broader risk of *tet*(G) acquisition.

### The ARGs in *B. bronchiseptica* and *B. trematum*

In *B. bronchiseptica*, we identified ARGs such as *sul1*, *bla*_OXA-2_, *aph(3'’)-Ib*, and *aph(6)-Id* (Figure S2A). These genes co-occurred within a 58 kb nucleotide sequence detected in four genomes, one of which constitutes a complete plasmid, designated *B. bronchiseptica* plasmid R906, uploaded by Petrovski, S. in 2020. In addition to ARGs, this plasmid harbours genes conferring resistance to heavy metals, such as *merA*, *merD*, and *merE*. The plasmid belongs to the IncP1 replicon type, a broad-host-range plasmid widely distributed among Gram-negative bacteria.^14^ IncP1 plasmids have been shown to transfer and stably persist in *B. pertussis* under laboratory conditions.^15^ Moreover, a naturally occurring IncP1-type plasmid was reported in *B. pertussis* in 2002.^16^ Comparative analysis of plasmid-derived sequences from *B. bronchiseptica* revealed a highly similar plasmid backbone, suggesting that the *Bordetella* genus may rely on IncP1-type plasmids for acquiring resistance phenotypes.

Among the 14 *B. trematum* genomes analysed, two strains carried ARGs. One strain harboured *tet*(31) and *sul2*, while the other carried *sul1*, *tet*(G), *floR*, *aadA1*, and *cmlA1* (Figure S2B). Comparative analysis with strain CBM01 revealed that the region containing ARGs in these two *B. trematum* genomes was integrated at the same chromosomal locus. In *B. avium*, HGT events were identified at two distinct chromosomal sites. Compared with the reference genome JBBA, strain BA8 acquired *sul2* and *tet*(A) at separate loci (Figure S2C), suggesting independent HGT events.

### Phylogenetic analysis based on core genome SNPs

**Figure 3.**
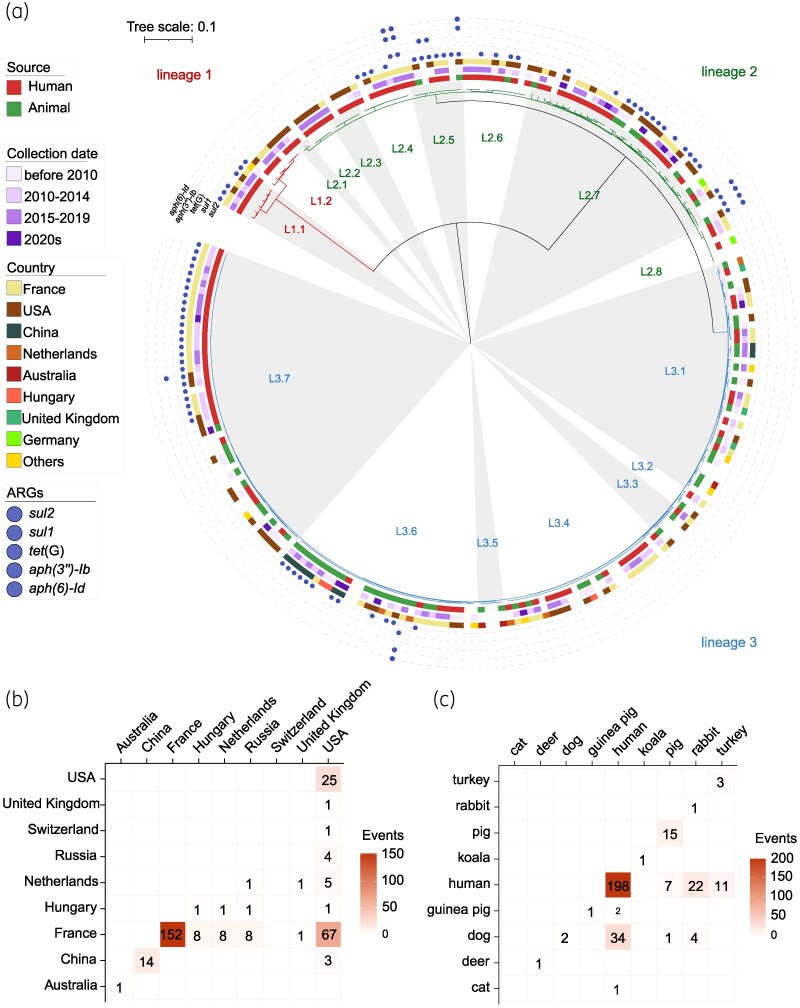
Phylogenetic Analysis and transmission characteristics of *B. bronchiseptica*. (a) Maximum likelihood phylogenetic tree of 238 *B. bronchiseptica* isolates. Scale bar represents nucleotide substitutions per site. (b) Heatmap depicting the number of inferred direct transmission events (SNP threshold < 5) between countries. Colour intensity is proportional to the number of events and does not account for the sample size per country. (c) Heatmap depicting the number of inferred direct transmission events (SNP threshold < 5) between host sources. Colour intensity is proportional to the number of events and does not account for the sample size per source.

To elucidate the dissemination of resistance determinants within the *Bordetella* genus, we performed a core-genome SNP-based phylogenetic analysis on 238 *B. bronchiseptica* isolates exhibiting the highest resistance-gene carriage. Bayesian clustering resolved the population into three principal lineages (lineage 1, lineage 2 and lineage 3; see Figure 3a), which were further subdivided into 17 sub-lineages. All isolates in lineage 1 originated from human hosts, whereas lineages 2 and 3 encompassed human, animal and environmental sources, indicating greater potential for cross-ecological transmission. No temporal clustering was observed among the three lineages, as isolates collected in different years exhibited comparable genetic distances, consistent with the known genomic conservation and low mutation rate of *B. bronchiseptica*. Among the 17 sub-lineages, strains from France and the United States were widely distributed, while Chinese isolates were predominantly assigned to sub-lineage L3.6, with a minority in L3.1. Analysis of resistance gene prevalence revealed that *sul2*, the most prevalent determinant, was unevenly distributed and particularly enriched in lineages L1.1, L2.4–L2.7, L3.6, and L3.7. Each of these sub-lineages includes human-derived isolates and thus represents a potential high-risk group. In addition, multidrug resistant isolates carrying multiple resistance genes were chiefly confined to lineage 2.

### Quantification of transmission events using SNP thresholds

To quantify and compare transmission dynamics, single SNP differences were classified as clonal transmission (SNPs < 5) or close/related transmission (5 ≤ SNPs < 20). Across all isolates, 492 clonal transmission events were identified, of which 304 had unambiguous information on country of isolation and host (Table S2). Among these 304 events, international transmission was frequent. Events involving France were the most numerous (152 domestic; 92 international). In contrast, for events involving the USA, international transmission constituted the majority (76.6%, 82/107) (Figure 3b). Numerous human-human events were detected, which may partly explain the high incidence of international spread. Cross-host transmission accounted for 27.0% (82/304) of events. Human-animal transmission comprised 77 cases, with human-dog (*n* = 34) and human-rabbit (*n* = 22) being the most common (Figure 3c). Expanding the threshold to SNPs < 20 uncovered additional transmission events, notably between the United States, China and France, and a marked increase in cross-host spread (Figure S3A). Human-pig close transmissions were most frequent (95 events) (Figure S3B).

### Domestic versus international transmission patterns in France and the United States

**Figure 4.**
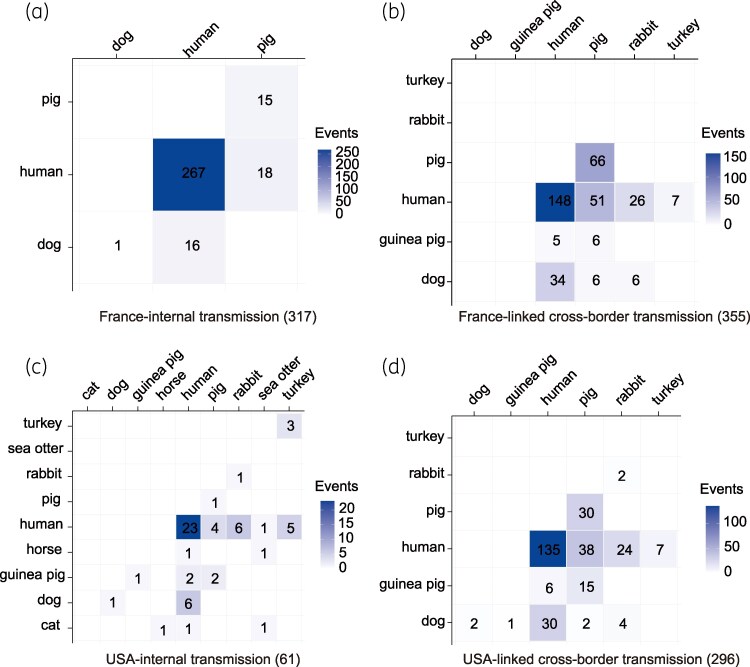
Transmission patterns centred on France and the USA with source composition of transmission events (SNPs < 20). (a) Local transmission events within France. (b) International transmission events linked to France. (c) Local transmission events within the USA. (d) International transmission events linked to the USA.

Using SNPs < 20 as the criterion, domestic and international transmission events were tallied for the two principal source countries. France: 317 domestic versus 355 international events. Domestic spread was overwhelmingly human-human (84.2%, 267/317), with additional transmission observed between humans and dogs or pigs (Figure 4a). International events involved a broader host spectrum, though humans remained predominant (76.3%, 271/355); pigs, dogs and rabbits also featured prominently (Figure 4b). United States: 61 domestics (Figure 4c) versus 296 international events (Figure 4d), a pattern sharply contrasting with France. While domestic spread was predominantly human-human, the international profile resembled that of France, with human-centric transmission accompanied by significant pig, dog and rabbit involvement.

In both countries, international events outnumbered domestic ones—particularly in the United States. Dogs represent a principal host for *B. bronchiseptica*,^17^ and both France and the United States are major players in the global pet-dog trade.^18^ The international movement of dogs infected with specific clonal lineages could represent one of several possible contributors to international clonal dissemination. In the U.S. network, pet-trade-mediated human-dog/rabbit transmissions are frequently observed and may constitute a pathway through which international spread can exceed local transmission.

These findings indicate that human activity and the international movement of animals—particularly the pet-dog trade—are important routes for *B. bronchiseptica* dissemination, occasionally exerting greater influence than domestic transmission networks. This underscores the urgent need for stringent international pathogen control measures and enhanced international collaboration to prevent the spread of animal respiratory pathogens.

The number of *Bordetella* genomes analysed in this study is relatively limited and unevenly distributed across species. *B. bronchiseptica* is notably overrepresented, while genome sequences for other species remain scarce in public databases, likely contributing to the lower absolute number of ARGs detected in those taxa. These disparities may reflect both genuine differences in species prevalence and biases in genomic surveillance. To overcome these limitations, broader genome sequencing efforts across the genus are needed. We advocate for increased investment in this area, as integrating future datasets will be critical for more comprehensive assessments of AMR in *Bordetella*.

### Conclusions

This work delivers the first comprehensive survey of acquired ARGs in non-*B. pertussis Bordetella* species, revealing ten ARG classes—including sulphonamide, tetracycline, aminoglycoside and β-lactam determinants—distributed across *B. bronchiseptica*, *B. trematum* and *B. avium*. Phylogenetic and transmission analyses demonstrate that, despite overall genomic conservation, *B. bronchiseptica* can integrate mobile elements carrying multiple ARGs, with evidence of clonal expansion and international, cross-host spread. The identification of plasmid- and transposon-mediated transfer underscores a risk for future ARG acquisitions in clinical and environmental *Bordetella*. These findings highlight the necessity for sustained, global genomic surveillance and collaboration to forestall the emergence and dissemination of resistant *Bordetella* strains.

## Supplementary Material

dkaf418_Supplementary_Data

## Supplementary data

Figures S1 to S3 and Tables S1 and S2 are available as Supplementary data at *JAC* Online.
