# Supplementary material for: Acquired antimicrobial resistance genes in Bordetella Species: a global genomic analysis
Source: J Antimicrob Chemother. 2025 Dec 1;81(1):dkaf418. doi: 10.1093/jac/dkaf418 (PMC12972672; doi:10.1093/jac/dkaf418)
Supplement: dkaf418_Supplementary_Data [file dkaf418_supplementary_data.zip › supplementary figure S1-S3.docx]

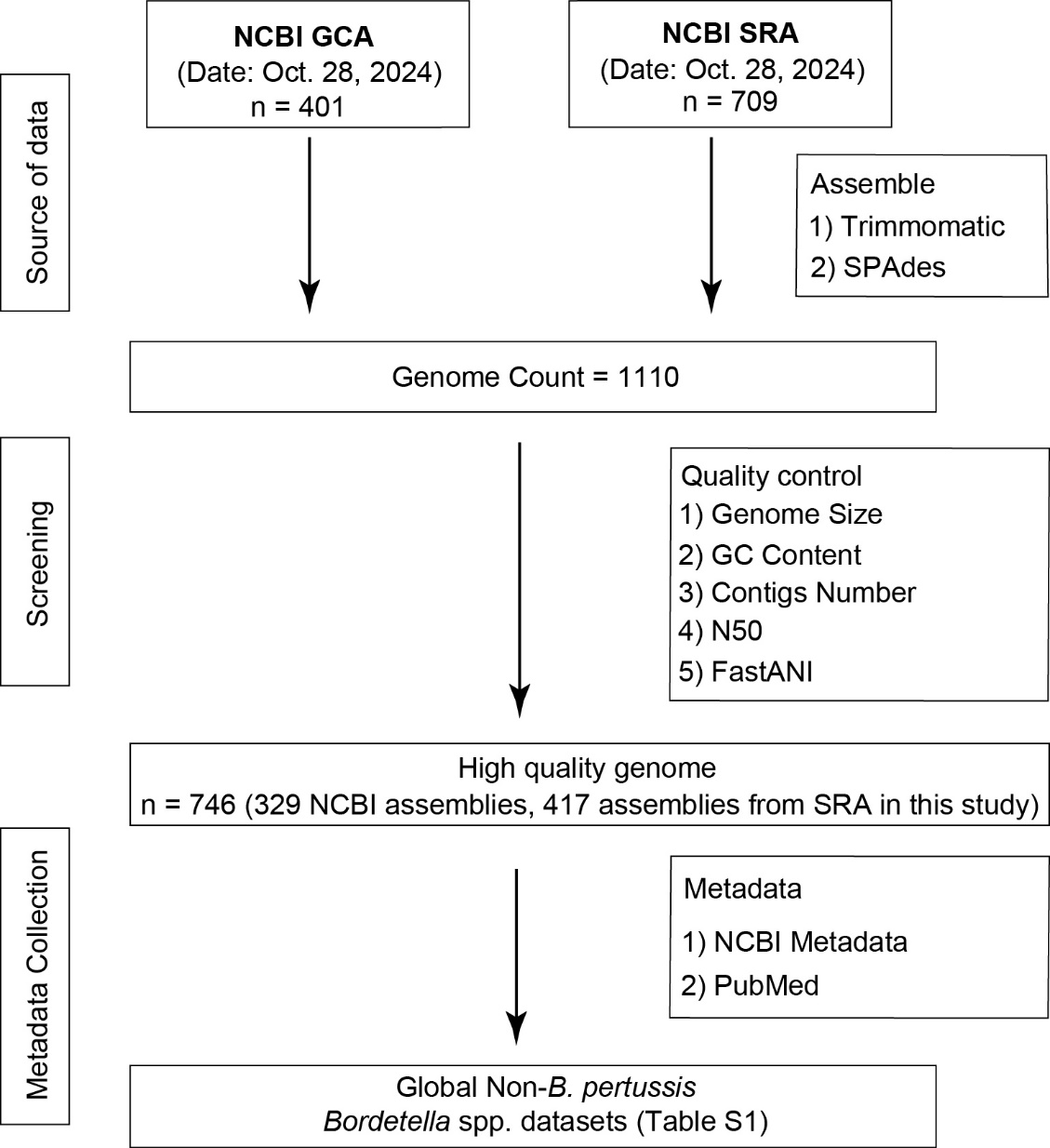


**Figure S1.** The quality control process of the genomes of the genus *Bordetella* other than *B. pertussis.*


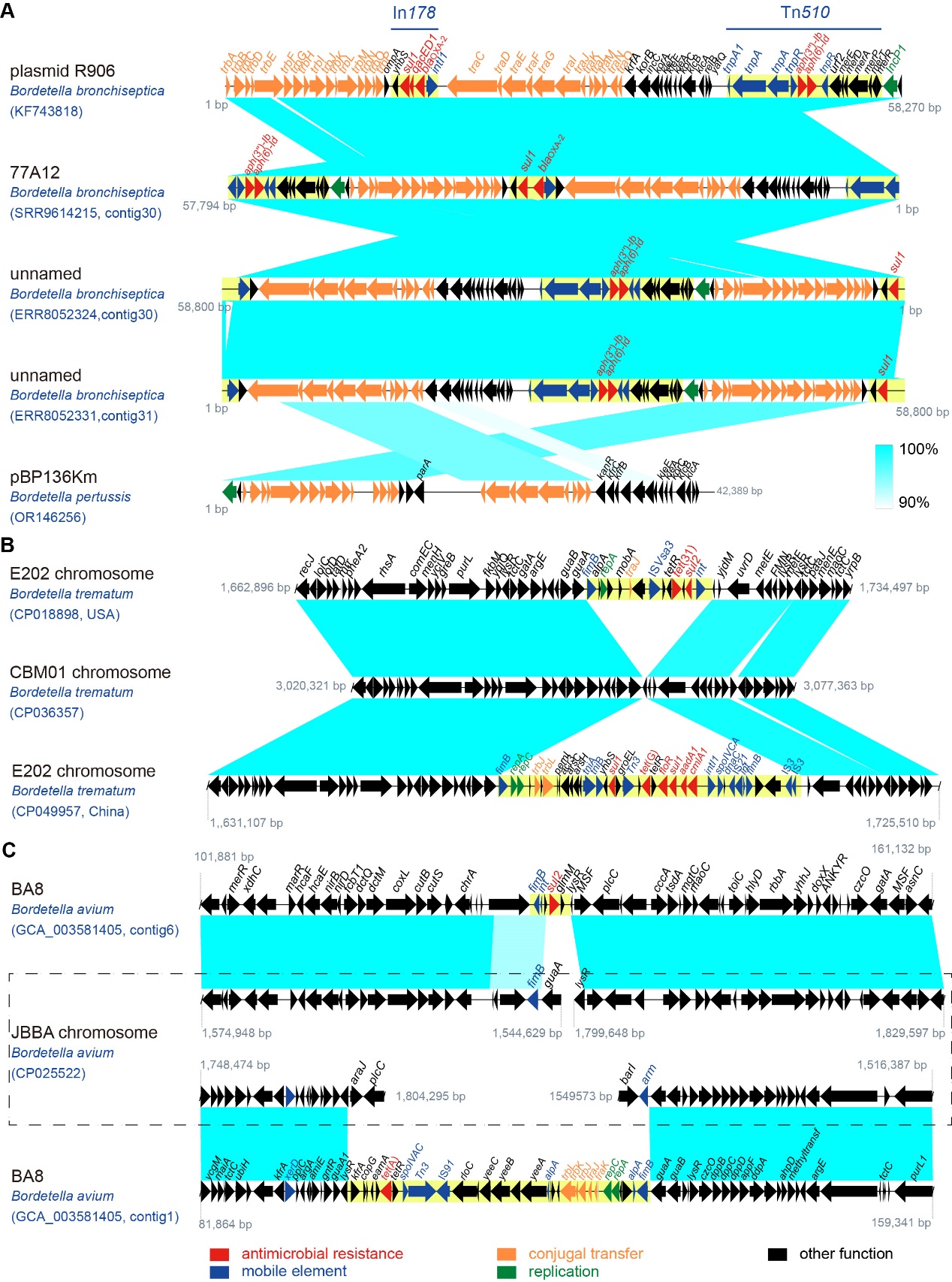


**Figure S2.** Genetic environment analysis of acquired resistance genes in *B. bronchiseptica* (**A**), *B. trematum* (**B**), and *B. avium* (**C**). In (B), the two strains E202 are completely different strains, one from China and the other from the United States.


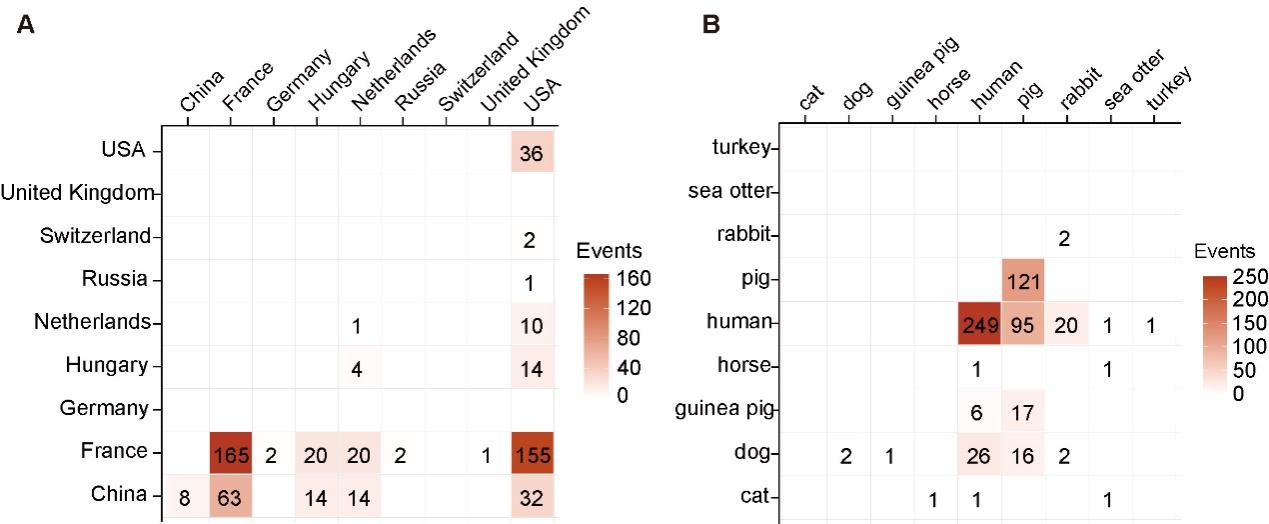


**Figure S3.** Close related transmission characteristics of *B. bronchiseptica.* (**A**) Heatmap depicting the number of inferred direct transmission events (5≤ SNPs < 20) between countries. Color intensity is proportional to the number of events and does not account for the sample size per country. (**B**) Heatmap depicting the number of inferred direct transmission events (5≤ SNPs < 20) between host sources. Color intensity is proportional to the number of events and does not account for the sample size per source.
